# The Effect of a Single Bout of Exercise to Volitional Exhaustion Under Moderate Normobaric Hypoxia on the Kinetics of Cardiac Biomarkers in Trained and Untrained Men

**DOI:** 10.3390/ijms27125234

**Published:** 2026-06-09

**Authors:** Miłosz Czuba, Kamila Płoszczyca, Adam Niemaszyk, Natalia Grzebisz-Zatońska, Małgorzata Chalimoniuk, Józef Langfort, Katarzyna Kaczmarczyk, Robert Gajda

**Affiliations:** 1 Faculty of Rehabilitation, Józef Piłsudski University of Physical Education in Warsaw, 00-968 Warsaw, Poland; kamila.ploszczyca@awf.edu.pl (K.P.); adam.niemaszyk@awf.edu.pl (A.N.); natalia.grzebisz@awf.edu.pl (N.G.-Z.); katarzyna.kaczmarczyk@awf.edu.pl (K.K.); 2 Department of Physical Education and Health in Biala Podlaska, Faculty in Biala Podlaska, Jozef Pilsudski University of Physical Education in Warsaw, 21-500 Biala Podlaska, Poland; m.chalimoniuk@awf.edu.pl; 3 Department of Sports Theory, The Jerzy Kukuczka Academy of Physical Education, 40-065 Katowice, Poland; j.langfort@awf.katowice.pl; 4 Center for Sports Cardiology at the Gajda-Med Medical Center in Pultusk, 06-102 Pultusk, Poland; gajda@gajdamed.pl

**Keywords:** cardiac biomarkers, normobaric hypoxia, volitional exhaustion, cardiac troponin, trained athletes

## Abstract

Post-exercise release of cardiac biomarkers reflects physiological adaptations of the myocardium to exercise; however, data on their kinetics after exhaustive exercise under hypoxia remain scarce. We determined the kinetics of cardiac biomarker changes following a single bout of exercise to volitional exhaustion under normoxia and moderate normobaric hypoxia (2000 m and 3000 m a.s.l.) in trained (n = 12; VO_2max_ 64.2 ± 2.9 mL·kg^−1^·min^−1^) and untrained (n = 12; VO_2max_ 44.1 ± 7.4 mL·kg^−1^·min^−1^) men. Participants performed a graded exercise test (GXT) followed by a constant-workload exercise test (CXT) at the lactate threshold under three conditions (FiO_2_ = 20.9%, 16.5%, 14.4%). Venous blood was sampled at rest, immediately post-exercise, and at 2, 6, and 24 h of recovery for determination of cardiac troponin T (cTnT) and I (cTnI), myoglobin (Mb), creatine kinase MB isoform (CK-MB), heart-type fatty acid-binding protein (H-FABP), ischemia-modified albumin (IMA), and N-terminal pro-B-type natriuretic peptide (NT-proBNP) by ELISA. Exhaustive exercise induced significant elevations in all biomarkers, peaking at 2–6 h post-exercise and largely returning to resting values by 24 h. Moderate normobaric hypoxia did not augment the cardiac biomarker response; rather, it attenuated the increases in Mb, NT-proBNP, and IMA, likely due to earlier peripheral fatigue and lower absolute mechanical work. The inhibitory effect of hypoxia on cTnI release was observed exclusively in trained men, suggesting an interaction between training-related cardiac adaptations and the hypoxic stimulus. These findings support the safety of high-intensity exercise at simulated altitudes of 2000–3000 m a.s.l.

## 1. Introduction

Cardiac biomarker testing is commonly used to diagnose myocardial damage [1]. In contrast to pathophysiological changes, the post-exercise release of cardiac enzymes represents a physiological response of the body, which may reflect the effectiveness of the training stimulus and indicate morphological and functional adaptations of the myocardium to physical exercise [2,3,4]. Previous studies have demonstrated that both prolonged and short-term high-intensity exercise lead to transient changes in cardiac biomarker concentrations in the blood of athletes [4,5,6,7,8,9]; however, the magnitude and nature of these changes differ depending on the type and duration of the activity.

The mechanism governing the post-exercise release of enzymes has yet to be fully elucidated. The main causes of this response, apart from potential damage to the cardiomyocytes, include the release of enzymes from the cytosol resulting from increased membrane permeability, rather than from cardiomyocyte necrosis or from skeletal muscle injury when biomarkers with a low cardiac specificity were used [5,8,10,11,12]. In most cases, biomarkers return to baseline 24 h post-exercise, and studies that employed electrocardiography or diagnostic imaging techniques [12,13,14] revealed no concurrent signs of cardiac damage.

The release of cardiac biomarkers is thought to be influenced not only by exercise but also by the environmental conditions under which exercise is performed, including hypoxic environments [15,16]. One of the key metabolic effects of hypoxia exposure on the heart is a shift in the energy substrate preference of cardiomyocytes: under oxygen deficiency, the heart increases its reliance on glucose as a substrate for ATP production at the expense of fatty acids [17,18]. Although such a metabolic response is adaptive in nature, animal model studies indicate that a prolonged or augmented increase in myocardial glucose utilization may promote adverse structural remodeling [19,20]. At the hemodynamic level, short-term hypoxia exposure activates the sympathetic nervous system, resulting in increased heart rate, augmented cardiac output, and elevated pulmonary vascular resistance [21]. The right ventricle (RV) appears to be particularly vulnerable to hypoxia: hypoxic pulmonary vasoconstriction and the resulting elevation in pulmonary arterial pressure (PAP) directly increase its afterload [22,23].

Despite growing interest in hypoxia in the context of exercise physiology, as well as its application for training and therapeutic purposes [24], the literature still lacks sufficient data regarding the effects of exercise combined with hypoxic conditions on myocardial status. Our recent echocardiographic studies, conducted immediately post-exercise in both trained and untrained individuals, showed that hypoxic conditions (~3000 m a.s.l., FiO_2_ = 14.4%) did not augment post-exercise impairment of left ventricular (LV) function. Notably, in the athlete group, exercise in hypoxia elicited a marginally smaller deterioration in LV Global Longitudinal Strain (GLS) compared to exercise under normoxic conditions [25]. Analogous findings were observed for the right ventricle (RV): hypoxia did not augment post-exercise changes in RV systolic function in either group and its effect at rest was limited to minor hemodynamic alterations, observed exclusively in untrained individuals [26]. Furthermore, in our previous pilot study we showed that moderate normobaric hypoxia does not exacerbate the acute response of cardiac biomarkers to high-intensity exercise [27]. In that study, trained athletes showed a significant increase in cardiac troponin T (cTnT), myoglobin (Mb), and heart-type fatty acid-binding protein (H-FABP) concentrations following interval exercise, yet without significant differences between conditions (normoxia vs. normobaric hypoxia FiO_2_ = 15.5%; ~2500 m). It should be noted, however, that one limitation of that study was that biomarkers were assessed exclusively immediately after exercise, without evaluation of their time course kinetics. Therefore, the question of the kinetics of cardiac biomarker changes following exhaustive exercise remains unresolved, as does the question of whether varying the intensity of the hypoxic stimulus modifies the cardiac biomarker response to exercise.

The aim of the present study was to determine the kinetics of cardiac biomarker changes in response to a single bout of exercise performed to volitional exhaustion under normoxia and moderate normobaric hypoxia corresponding to 2000 m and 3000 m a.s.l. in trained and untrained men. Based on the findings of previous studies, we formulated the following hypotheses: (1) a single bout of exercise to volitional exhaustion under normobaric hypoxia will not elicit a greater cardiac biomarker response compared to normoxia; (2) the observed changes will be of a similar nature in trained and untrained men; (3) the severity of hypoxia will influence the kinetics of the investigated cardiac biomarkers.

## 2. Results

In both groups (UT and T), exercise to exhaustion induced significant changes in the concentrations of all cardiac biomarkers studied (cTnT, cTnI, Mb, H-FABP, CK-MB, IMA and NT-proBNP) under all experimental conditions (N, H2000, H3000), except for cTnI under H3000 in the T group. Detailed results of the Friedman ANOVA are presented in Table 1 and Table 2.

### 2.1. cTnT—Cardiac Troponin T

In the UT group, cTnT concentrations increased significantly (*p* < 0.05) immediately after exercise under all three experimental conditions (+29.9% under N, +20.2% under H2000, +12.9% under H3000). At 2 h post-exercise, a further significant increase (*p* < 0.05) was observed under all conditions (+57.6% in N, +29.2% under H2000, +26.7% under H3000) relative to resting values. At 6 h post-exercise, cTnT concentrations declined; however, values remained significantly elevated (*p* < 0.05) above resting levels under normoxic conditions (+40.9%) and under H2000 (+21.5%). Under H3000, cTnT changes at 6 h no longer reached statistical significance. At 24 h post-exercise, a significantly elevated (*p* < 0.05) cTnT concentration (+15.8%) was observed exclusively under N.

In the T group, cTnT concentrations likewise increased significantly (*p* < 0.05) immediately after exercise under all three conditions (+27.2% under N, +22.0% under H2000, +16.7% under H3000). At 2 h post-exercise, a further significant increase (*p* < 0.05) in cTnT was recorded under all conditions (+32.9% under N, +27.7% under H2000, +13.2% under H3000). At 6 h post-exercise, cTnT concentrations remained significantly elevated (*p* < 0.05) under all conditions (+31.6% under N, +14.8% under H2000, +14.1% under H3000). At 24 h post-exercise, no significant differences from resting values were observed in the T group.

### 2.2. cTnI—Cardiac Troponin I

In the UT group, cTnI concentrations did not change significantly immediately after exercise under any condition. Significant increases (*p* < 0.05) in circulating cTnI were first observed at 2 h and 6 h post-exercise under all experimental conditions (N: +22.2% at 2 h, +19.2% at 6 h; H2000: +18.3% at 2 h, +22.2% at 6 h; H3000: +19.6% at 2 h, +19.7% at 6 h). At 24 h post-exercise, no significant differences from resting values were observed.

In the T group, cTnI concentrations did not change significantly either immediately or at 2 h after exercise under H2000 and H3000. A significant increase (*p* < 0.05) in cTnI was recorded at 2 h post-exercise exclusively under N (+7.9%). At 6 h post-exercise, significant elevations (*p* < 0.05) were observed under N (+20.6%) and H2000 (+14.1%). Changes in cTnI under H3000 did not reach statistical significance at any time point. Additionally, at 24 h post-exercise, a significantly elevated cTnI concentration (+12.5%, *p* < 0.05) persisted under N.

### 2.3. Mb—Myoglobin

In the UT group, a significant increase (*p* < 0.05) in Mb concentration was observed immediately after exhaustive exercise under all experimental conditions (+36.2% under N, +21.4% under H2000, +21.2% under H3000). At 2 h post-exercise, a further significant increase (*p* < 0.05) was recorded under all conditions relative to resting values (+64.3% under N, +34.7% under H2000, +30.2% under H3000). Mb concentrations also remained significantly elevated (*p* < 0.05) at 6 h post-exercise under all conditions (+21.6% under N, +19.9% under H2000, +25.8% under H3000). Notably, at 24 h post-exercise, a significantly elevated (*p* < 0.05) Mb concentration (+18.2%) was observed exclusively under N in the UT group.

Similar changes were observed in the T group, in which Mb concentrations likewise increased significantly (*p* < 0.05) immediately after exercise under all conditions (+11.3% under N, +13.9% under H2000, +8.6% under H3000). At 2 h post-exercise, a further significant increase (*p* < 0.05) was recorded under all conditions (+40.8% under N, +30.3% under H2000, +16.6% under H3000). Mb concentrations remained significantly elevated (*p* < 0.05) at 6 h post-exercise under all conditions relative to resting values (+43.9% under N, +17.2% under H2000, +13.4% under H3000). At 24 h post-exercise, no significant differences from resting values were observed in the T group.

### 2.4. H-FABP—Heart-Type Fatty Acid-Binding Protein

In the UT group, H-FABP concentrations increased significantly (*p* < 0.05) immediately after exercise under N (+19.5%), H2000 (+10.7%) and H3000 (+7.1%). At 2 h post-exercise, significant increases (*p* < 0.05) were recorded under all three conditions (+32.7% under N, +18.7% under H2000, +15.9% under H3000). At 6 h post-exercise, significantly elevated (*p* < 0.05) values persisted under H2000 (+17.9%). At 24 h post-exercise, no significant changes were observed.

In the T group, H-FABP concentrations increased significantly (*p* < 0.05) immediately after exercise under N (+21.1%) and H2000 (+7.2%). No significant changes were observed immediately post-exercise under H3000. At 2 h post-exercise, significant increases (*p* < 0.05) were recorded under all three conditions (+34.4% under N, +20.9% under H2000, +26.0% under H3000). At 6 h post-exercise, significantly elevated (*p* < 0.05) values were maintained exclusively under N (+25.8%). At 24 h post-exercise, no significant changes in H-FABP were observed in this group.

### 2.5. CK-MB—Creatine Kinase MB Isoform

In the UT group, CK-MB concentrations increased significantly (*p* < 0.05) immediately after exercise under N (+7.1%) and H3000 (+10.1%). No significant change was observed immediately post-exercise under H2000. At 2 h post-exercise, significant increases (*p* < 0.05) were present under all conditions (+21.5% under N, +23.2% under H2000, +29.1% under H3000). At 6 h post-exercise, CK-MB concentrations continued to increase significantly (*p* < 0.05) under all conditions (+27.6% under N, +28.9% under H2000, +30.3% under H3000). At 24 h post-exercise, no significant differences from resting values were observed.

Changes in CK-MB in the T group followed a similar pattern. CK-MB concentrations increased significantly (*p* < 0.05) immediately after exhaustive exercise under all conditions (+7.5% under N, +10.1% under H2000, +9.4% under H3000). Significant elevations (*p* < 0.05) were also observed at 2 h post-exercise under all conditions (+18.8% under N, +15.4% under H2000, +15.9% under H3000). Significant increases (*p* < 0.05) were maintained under all conditions at 6 h post-exercise (+25.8% under N, +19.6% under H2000, +23.9% under H3000). At 24 h post-exercise, no significant differences from resting values were observed.

### 2.6. NT-proBNP—N-Terminal Pro-B-Type Natriuretic Peptide

In the UT group, NT-proBNP concentrations increased significantly (*p* < 0.05) immediately after exercise under all experimental conditions (+16.2% under N, +20.1% under H2000, +11.8% under H3000). A further significant increase (*p* < 0.05) relative to resting values was likewise observed at 2 h post-exercise under all conditions (+30.9% under N, +27.3% under H2000, +14.7% under H3000). These elevations were maintained (*p* < 0.05) at 6 h post-exercise (+11.1% under N, +22.7% under H2000, +13.7% under H3000). At 24 h post-exercise, a significantly elevated (*p* < 0.05) NT-proBNP concentration (+11.5%) was observed exclusively under N conditions.

Notably, NT-proBNP concentrations in the T group did not change significantly immediately after exercise under any condition. A significant increase (*p* < 0.05) was first detected at 2 h post-exercise under all conditions (+24.6% under N, +16.6% under H2000, +17.5% under H3000). At 6 h post-exercise, significantly elevated (*p* < 0.05) values were maintained exclusively under N (+15.7%). At 24 h post-exercise, no significant changes in NT-proBNP were observed under any condition.

### 2.7. IMA—Ischemia-Modified Albumin (IMA)

In the UT group, IMA concentrations increased significantly (*p* < 0.05) immediately after exercise under N (+40.7%), H2000 (+72.0%), and H3000 (+89.5%). At 2 h post-exercise, significant increases (*p* < 0.05) were observed under all three conditions (+50.0% under N, +78.9% under H2000, and +125.0% under H3000). At 6 h and 24 h post-exercise, significantly elevated values (*p* < 0.05) persisted under all conditions (at 6 h: +58.7% under N, +82.0% under H2000, and +103.2% under H3000; at 24 h: +42.1% under N, +41.6% under H2000, and +43.1% under H3000).

In the T group, IMA concentrations increased significantly (*p* < 0.05) immediately after exercise under N (+22.4%), H2000 (+20.3%), and H3000 (+34.1%). At 2 h post-exercise, significant increases (*p* < 0.05) were recorded under all three conditions (+40.2% under N, +47.9% under H2000, and +40.4% under H3000). At 6 h post-exercise, significantly elevated values (*p* < 0.05) persisted under all conditions (at 6 h: +48.0% under N, +48.7% under H2000, and +47.6% under H3000). At 24 h post-exercise, a significantly elevated IMA concentration (*p* < 0.05; +14.1%) was observed exclusively under N.

### 2.8. Twenty-Four-Hour Post-Exercise Summary

It is noteworthy that at 24 h post-exercise, most of the studied biomarkers had returned to resting values in both groups. In the T group, cTnI and IMA concentrations remained significantly elevated (*p* < 0.05) under N (+12.5% and +14.1%, respectively). In the UT group, three markers likewise remained significantly elevated (*p* < 0.05) under N: Mb (+18.2%), cTnT (+15.8%), NT-proBNP (+11.5%), and IMA (42.1%). Additionally, IMA remained significantly elevated (*p* < 0.05) in H2000 (41.6%), and H3000 (43.1%) Concentrations of cTnI, CK-MB, and H-FABP did not differ significantly from resting values at 24 h under any condition in the UT group.

### 2.9. Percentage Changes (Δ%) of the Cardiac Biomarkers Across Groups and Study Conditions

A one-way Friedman ANOVA conducted on percentage changes (Δ%) in the biomarkers examined between experimental conditions revealed statistically significant between-condition differences in both groups.

In the UT group, significant between-condition differences were demonstrated for Δ%Mb immediately post-exercise (χ^2^(2) = 8.000; *p* = 0.018) and at 2 h after exercise to exhaustion (χ^2^(2) = 8.166; *p* = 0.016). Significant between-condition differences were also found for Δ%cTnT at 2 h post-exercise (χ^2^(2) = 7.166; *p* = 0.027). Similarly, a statistically significant between-condition difference was observed for Δ%NT-proBNP at the same time point (χ^2^(2) = 11.872; *p* = 0.002).

In the T group, a comparable pattern was observed. Significant between-condition differences were found for Δ%Mb immediately post-exercise (χ^2^(2) = 8.166; *p* = 0.016) and at 2 h post-exercise (χ^2^(2) = 12.166; *p* = 0.002). Additionally, a statistically significant between-condition difference was demonstrated for Δ% NT-proBNP at 6 h post-exercise (χ^2^(2) = 11.166; *p* = 0.003) and Δ%IMA at 24 h post-exercise (χ^2^(2) = 6.500; *p* = 0.038).

**Figure 1:**
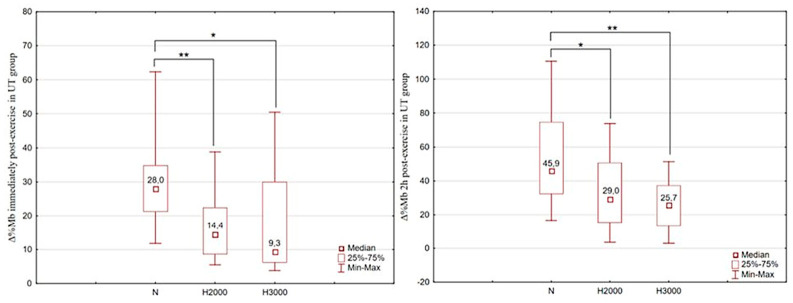
Percentage changes (Δ%) of Mb immediately and at 2 h post-exercise in the UT group. N—normoxia, H2000 and H3000—normobaric hypoxia corresponding to 2000 m and 3000 m. ** *p* < 0.01, * *p* < 0.05—significant differences between conditions.

**Figure 2:**
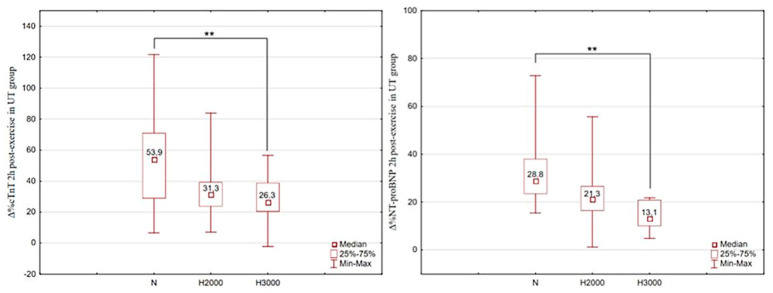
Percentage changes (Δ%) of cTnT and NT-proBNP at 2 h post-exercise in the UT group. N—normoxia, H2000 and H3000—normobaric hypoxia corresponding to 2000 m and 3000 m. ** *p* < 0.01—significant differences between conditions.

**Figure 3:**
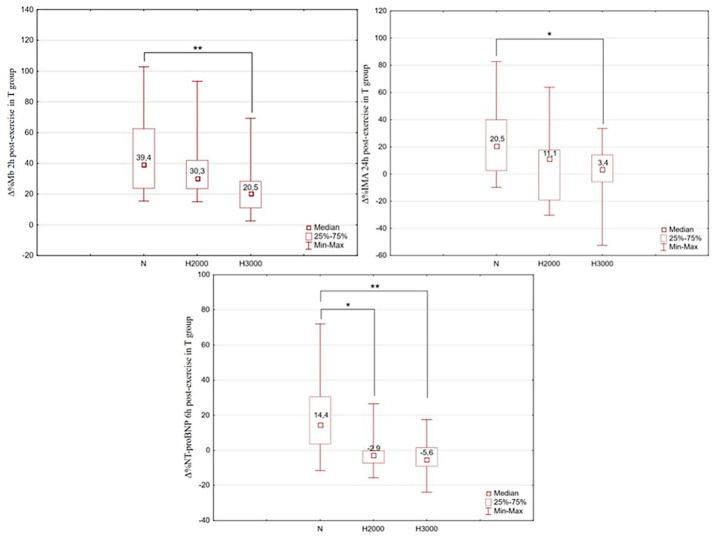
Percentage changes (Δ%) of Mb at 2 h post-exercise, NT-proBNP at 6 h post-exercise, and IMA at 24 h post-exercise in the T group. N—normoxia, H2000 and H3000—normobaric hypoxia corresponding to 2000 m and 3000 m. ** *p* < 0.01, * *p* < 0.05—significant differences between conditions.

Post hoc analysis using the Wilcoxon signed-rank test in the UT group revealed significantly (*p* < 0.05) lower Δ%Mb under both H2000 and H3000 conditions relative to N immediately post-exercise (Figure 1). A similar pattern was observed at 2 h post-exercise, where Δ%Mb remained significantly (*p* < 0.05) lower under both hypoxic conditions (H2000 and H3000) compared to N (Figure 1). Furthermore, Δ%cTnT and Δ%NT-proBNP at 2 h post-exercise were significantly (*p* < 0.05) lower under H3000 relative to N (Figure 2). In the T group, a comparable pattern was observed—Δ%Mb at 2 h post-exercise was significantly (*p* < 0.05) lower under H3000 compared to N (Figure 3). Additionally, the Δ%NT-proBNP at 6 h post-exercise was significantly (*p* < 0.05) lower under H2000 and H3000 relative to N (Figure 3). Furthermore, the Δ%IMA at 24 h post-exercise was significantly (*p* < 0.05) lower under H3000 relative to N.

### 2.10. Changes in Mechanical Work, Heart Rate and Saturation of Hemoglobin During Exercise to Exhaustion Under Normoxia and Hypoxia

Friedman ANOVA revealed a significant effect of experimental conditions for mechanical work (W_mech_; UT: χ^2^(2) = 16.167; *p* < 0.001; T: χ^2^(2) = 6.000; *p* = 0.050), heart rate recorded at the end of exercise performed to exhaustion (HR_end_; UT: χ^2^(2) = 11.261; *p* = 0.004; T: χ^2^(2) = 10.500; *p* = 0.005), and hemoglobin saturation registered at the end of exercise (SpO_2_; UT: χ^2^(2) = 23.532; *p* < 0.001; T: χ^2^(2) = 20.667; *p* < 0.001). No significant differences in changes in blood lactate concentration in response to exercise (ΔLA) and changes in pH in response to exercise ΔpH were found in either group.

In the UT group, a significant reduction (*p* < 0.05) in W_mech_ was observed under H3000 compared to N (−18.6%) and compared to H2000, with no significant difference between N and H2000. In the T group, a significant reduction (*p* < 0.05) occurred exclusively under H3000 compared to N (−18.7%), while the difference under H2000 was non-significant. In all conditions, the T group performed significantly greater (*p* < 0.05) W_mech_ than the UT group (Table 3).

In the UT group, a significant reduction (*p* < 0.05) in HR_end_ was observed exclusively under H3000 compared to N (−4.6%), with no significant difference in H2000. In the T group, a significant reduction (*p* < 0.05) was found under both H2000 (−3.9%) and H3000 (−4.2%) compared to N, with no significant difference between hypoxic conditions (Table 3).

In both groups, a significant reduction (*p* < 0.05) in SpO_2_ was observed under H2000 (UT: −6.7%; T: −5.0%) and H3000 (UT: −11.7%; T: −10.4%) compared to N. Values in H3000 were significantly lower (*p* < 0.05) than under H2000. Under N, the UT group exhibited significantly higher SpO_2_ than the T group (*p* < 0.05), whereas differences between groups under hypoxic conditions were non-significant.

## 3. Discussion

The present study investigated the effect of moderate normobaric hypoxia on the kinetics of selected cardiac biomarkers in response to exercise to exhaustion in untrained and trained men. The principal finding is that moderate hypoxic conditions (3000 m) were associated with a significantly smaller increase in Mb and NT-proBNP concentrations at 2 h post-exercise in both groups. At 24 h post-exercise, the majority of biomarkers had returned to resting values in both groups, particularly following exercise in hypoxic conditions.

These findings highlight the importance of considering not only the environmental conditions but also the time course of biomarker assessment when interpreting exercise-induced cardiac responses. Accordingly, the responses of individual cardiac biomarkers are discussed below, with particular emphasis on their temporal patterns and the influence of experimental conditions and training status.

### 3.1. Myoglobin

In our study, Mb concentrations increased under all conditions (normoxia vs. hypoxia) following exercise in both untrained and trained men. In the UT group, elevated Mb concentrations persisted at 24 h post-exercise under normoxic conditions. Regardless of training status, Δ%Mb under hypoxic conditions followed a similar direction of change to normoxia, but the magnitude was markedly attenuated. Significantly lower Δ%Mb under hypoxia relative to normoxia was observed in both untrained (H2000 and H3000: immediately after and at 2 h post-exercise) and trained men (H3000 only: at 2 h post-exercise).

These findings are broadly consistent with earlier reports on the effects of exercise on post-exercise Mb concentrations. The post-exercise rise in Mb concentration is directly associated with sarcolemmal damage resulting from muscular activity, leading to dysfunction of the sodium–potassium pump and the release of various metabolites and intracellular proteins, including Mb, into the bloodstream [28], which in severe cases may result in exertional rhabdomyolysis [29]. Previous studies have shown that the Mb response to exercise is significantly influenced not only by training experience and participant age [30], but also by exercise duration [31]. Increases in Mb concentration have been reported immediately following high-intensity interval exercise of both an endurance [27,32] and resistance nature [33].

Early reports suggested that hypoxic exposure alone may contribute to elevated Mb concentrations [34]; however, more recent studies have shown that an additional stimulus in the form of physical exercise is necessary for such an increase to occur, and that the exposure period must exceed a single session [35]. Shave et al. [36] found no significant increase in Mb concentration in triathletes immediately following 120 min of cycling at an intensity approximating the anaerobic threshold, under either normoxic or hypoxic conditions (FiO_2_ = 15%). In our own previous study [27], using a closely comparable hypoxic stimulus (FiO_2_ = 15.5%, ~2500 m a.s.l.) and high-intensity interval exercise (90% VO_2max_), no significant differences between normoxia and hypoxia were found immediately post-exercise. The absence of measurements at later time points, however, precluded assessment of this marker’s kinetics during recovery.

The findings of the present study suggest that exercise performed under moderate hypoxic conditions leads to a smaller efflux of Mb into the bloodstream compared to normoxia, which may reflect a lesser degree of skeletal muscle and myocardial damage. In our view, the observed attenuation of Δ%Mb following exercise under hypoxic conditions is attributable primarily to the lower total mechanical work performed by participants, itself a consequence of two factors: (1) a lower absolute workload resulting from the determination of exercise intensity relative to the lactate threshold established under hypoxic conditions, and (2) a shorter time to volitional exhaustion [37,38]. The study protocol does not permit an unambiguous determination of whether the attenuated Mb increases reflect the effect of hypoxia per se or solely the lower total mechanical work performed.

A further interpretive limitation is the difficulty in definitively identifying the tissue source of Mb, which is critical for properly evaluating the effect of hypoxia on the myocardium. Nicholson et al. [39], studying rats subjected to a four-week training cycle, demonstrated an increase in Mb concentration exclusively in skeletal muscle, alongside a concurrent decrease in cardiac muscle. This effect was attributed to reduced activity of the CN/NFAT pathway in response to physical exercise. However, the mechanisms regulating Mb expression in response to exercise and training remain incompletely understood, and extrapolation of findings from animal models to humans is substantially limited. Given the low cardiac specificity of Mb as a cardiac marker, arising from its presence in both skeletal and cardiac muscle, interpretation of the observed changes requires particular caution and should be considered in conjunction with results for biomarkers of higher cardiac specificity.

### 3.2. Heart-Type Fatty Acid-Binding Protein

Heart-type fatty acid-binding protein (H-FABP) is among the cardiac biomarkers considered to have relatively high cardiac specificity. However, studies on the effects of physical exercise on H-FABP concentrations remain scarce. H-FABP is a cytoplasmic protein whose principal physiological function is the transport of fatty acids from the cell membrane to the mitochondria, where they undergo β-oxidation—the primary energy source for the myocardium [40]. Under conditions of myocardial ischemia, free fatty acids accumulate in both the plasma and cardiac tissue, adversely affecting cardiomyocyte function [41] and compromising cell membrane integrity, which consequently leads to the efflux of H-FABP into the extracellular space and bloodstream [42]. The fatty acid-binding capacity of H-FABP has been shown to be modulated by reactive oxygen species generated during ischemia and reperfusion, suggesting a role for H-FABP in protecting cardiac cells against oxidative stress [43]. Despite its predominant expression in the myocardium—which justifies its use as a cardiac-specific marker [44]—it should be noted that H-FABP is also present at lower concentrations in skeletal muscle [45], which limits its diagnostic specificity in the context of physical exercise.

In the present study, H-FABP concentrations increased following exercise, peaking at 2 h post-exercise before gradually returning toward baseline, reaching resting values by 24 h post-exercise under all conditions in both groups. These findings are broadly consistent with previously reported kinetics of H-FABP following physical exercise. Earlier studies demonstrated increases in H-FABP immediately following prolonged exercise under normoxic conditions [46,47]. In our own pilot study, we observed an increase in H-FABP concentration immediately after a single one-hour bout of high-intensity exercise under both normoxic and normobaric hypoxic conditions (~2500 m a.s.l.), with no significant differences between conditions [27].

It is worth noting that the H-FABP increases observed in the present study were markedly smaller than those reported following extreme endurance events such as a marathon [46], which may reflect a moderate degree of myocardial stress associated with the exercise protocol used. In both the pilot study [27] and the present work, peak H-FABP concentrations remained within the reference range for healthy individuals [48], and moderate normobaric hypoxia did not exacerbate post-exercise increases in H-FABP.

### 3.3. Ischemia-Modified Albumin (IMA)

Ischemia-modified albumin (IMA) is a form of human serum albumin that exhibits reduced ability to bind transition metal ions—primarily cobalt, copper, and nickel—at the N-terminal region of the molecule [49]. The principal mechanisms underlying IMA formation are considered to be structural damage to the N-terminal fragment of albumin by reactive oxygen species (ROS) generated during ischemia and reperfusion, and oxidative modification induced by the metabolic acidosis accompanying tissue hypoxia. Common to both mechanisms is a reduction in albumin’s affinity for transition metal ions [50,51].

Existing studies reveal a complex IMA response to physical exercise, with the response pattern appearing to depend on exercise type, intensity, and duration, as well as the training status of participants. Żebrowska et al. [52] found no significant increase in IMA in elite ultramarathon runners immediately following a 24-h race. Similarly, Lippi et al. [47] showed that IMA concentrations did not change immediately after, or at 3, 6, or 24 h following completion of a half-marathon in recreational runners. Other studies have reported a delayed increase in IMA concentrations, observed 24–48 h after endurance exercise, reflecting skeletal muscle ischemia and oxidative stress [53]. In our earlier work [54], we observed a small, non-significant decrease in IMA concentration immediately after a graded exercise test to exhaustion in athletes, followed by a significant increase of 15.9% at 30 min post-exercise. Transient decreases in IMA concentration during and immediately after exercise have also been reported elsewhere [11,53,55]. These observations have been explained primarily by hemoconcentration—the rise in albumin concentration caused by hemoconcentration leads to an apparent reduction in IMA values, as the excess albumin binds more cobalt, reducing its free pool [55].

Other studies, however, have revealed a contrasting response pattern. Memmedov et al. [56], in a study involving boxers and kickboxers, observed a significant increase in IMA immediately following a training session. A strong positive correlation between IMA and lactate concentration has also been demonstrated [57], suggesting that under conditions of high-intensity exercise, lactic acidosis may act as a direct modifier of albumin structure and influence blood IMA concentrations. The findings of the present study confirm that exhaustive high-intensity exercise leads to a significant increase in IMA concentrations in both athletes and untrained individuals. The IMA increase is evident immediately after high-intensity exercise under normoxia, and elevated IMA concentrations persist during the subsequent hours of recovery (at 2 h and 6 h), as well as at 24 h post-exercise.

Previous research has suggested that regular endurance training may influence resting IMA concentrations and the IMA response to exercise. Lippi et al. [58,59] showed that IMA concentrations in well-trained athletes were elevated relative to untrained individuals, which was interpreted as the effect of chronic, repeated skeletal muscle ischemia associated with intensive endurance training. Our results did not confirm elevated resting IMA concentrations in athletes, and the pattern of IMA changes following exercise under normoxia was similar in the UT and T groups.

Although hypoxia appears to be a potential stimulus for IMA formation [60,61,62,63], to our knowledge no studies have directly examined IMA changes in response to hypobaric or normobaric hypoxic exposure during exercise or training in healthy sedentary individuals or athletes. The only exception is our earlier study [54], in which we demonstrated an increase in IMA of 27.6% at 30 min following a graded exercise test to exhaustion, and of 19.4% immediately after a 30 km time trial, in well-trained male endurance athletes. Both exercise tests were performed under normobaric hypoxic conditions at a simulated altitude of 2000 m a.s.l. (FiO_2_ = 16.5%). In the present study, we confirmed that exercise under moderate hypoxia (H2000 and H3000) leads to significant increases in IMA concentrations in both untrained individuals and athletes. Importantly, however, hypoxic conditions did not augment the IMA response to exercise compared to normoxia. Furthermore, in the athlete group, IMA concentrations returned to resting values at 24 h post-exercise following exercise in hypoxia, whereas after exercise under normoxia they remained elevated; Δ%IMA at 24 h was significantly lower under H3000 than under normoxia. This difference should be attributed to the lower total mechanical work performed by athletes under hypoxic conditions, resulting from the earlier onset of peripheral fatigue, which led to earlier termination of exercise.

It should be noted that although IMA is recognized as a marker of early myocardial ischemia in acute coronary syndromes [49,51], skeletal muscle represents an important extracardiac source of IMA [53,59]. The low tissue specificity of IMA reduces its utility as a diagnostic tool for exercise-induced myocardial ischemia, and IMA results in athletes may not be straightforwardly interpretable using standard reference values derived from sedentary populations [55,59].

### 3.4. Cardiac Isoenzyme of Creatine Kinase (CK-MB)

CK-MB is another biomarker of relatively moderate cardiac specificity, whose changes in response to physical exercise under normoxic conditions have been fairly well-described in the literature. CK-MB is a cytoplasmic enzyme that catalyzes the reversible transfer of a phosphate group from ATP to creatine, playing a key role in energy metabolism in tissues with high and variable energy demands. In the myocardium, CK-MB accounts for approximately 25–30% of total CK activity, whereas in skeletal muscle its contribution is only 1–3% [64]. Nevertheless, despite this low proportion, high-intensity exercise engaging a large muscle mass can lead to a significant increase in plasma CK-MB concentrations—first documented in marathon runners, in whom values comparable to those seen in myocardial injury were found despite normal cardiac scintigraphy findings [65,66].

In the present study, exercise to exhaustion induced a statistically significant increase in CK-MB concentrations in both groups. In both untrained and trained men, CK-MB activity increased significantly under all three experimental conditions (N, H2000, H3000), both immediately after exercise and at 2 h and 6 h of recovery. At 24 h post-exercise, CK-MB activity returned to baseline values under all conditions in both groups, indicating the reversible nature of the observed changes. Notably, Δ%CK-MB did not differ significantly between conditions (normoxia vs. hypoxia) in either group, which clearly distinguishes this biomarker from Mb, whose post-exercise response was attenuated by hypoxia.

These findings are consistent with earlier studies on the CK-MB response to physical exercise. Increases in CK-MB activity have been reported during and following prolonged endurance exercise [67,68] (Son et al., 2015; Nie et al., 2011), short-duration high-intensity exercise [9,69], and resistance training [70]. In our pilot study, we observed a trend toward increased CK-MB activity (+31.1%) following high-intensity exercise under normoxia, whereas no significant changes were found under hypoxia [27]. In that same study, we also demonstrated that systematic high-intensity training under hypoxic conditions contributes to a reduction in resting CK-MB activity [27]. Interestingly, in the present study resting CK-MB concentrations did not differ between groups (untrained vs. trained). This finding is in partial contrast to the observations of López-López & Pareja-Galeano [71], who reported that resting CK-MB activity in physically active individuals is nearly twice as high as in age- and sex-matched sedentary individuals.

With regard to between-group differences, we observed that the peak CK-MB values reached during post-exercise recovery were lower in the trained group than in the untrained group, despite the trained individuals having performed significantly greater total mechanical work on each occasion. This may reflect adaptive reductions in skeletal muscle cell membrane permeability resulting from training, which limit enzyme efflux into the bloodstream even under greater absolute exercise loads. This phenomenon is well documented for total CK activity in trained individuals [71,72], although its specific relationship to CK-MB has not previously been described.

In the present study, an enzyme-linked immunosorbent assay (ELISA) was used, which—unlike the widely used immunoinhibition method, which is susceptible to interference from the mitochondrial CK isoenzyme [73]—offers greater analytical specificity for CK-MB, thereby reducing the risk of falsely elevated results in the absence of true cardiomyocyte injury. Nevertheless, given the presence of CK-MB in skeletal muscle and the fact that this marker has largely been superseded by cardiac-specific troponins in clinical diagnostics [74], the changes in CK-MB concentrations observed in the present study should be interpreted with caution, as a reflection of the combined skeletal muscle and myocardial response to physical exercise.

### 3.5. Troponins (cTnT and cTnI)

We found that the post-exercise responses of cTnT and cTnI differed, both in terms of the temporal pattern of concentration changes and in relation to the experimental conditions. In both groups, cTnT concentrations increased significantly immediately after exercise under all conditions, peaking at 2 h post-exercise. At 6 h of recovery, cTnT remained elevated before returning to baseline at 24 h. Two exceptions were noted in the UT group: 1) under normoxia, cTnT remained significantly elevated at 24 h post-exercise; and 2) under H3000, cTnT no longer differed significantly from baseline as early as 6 h post-exercise. This observation suggests that in untrained individuals, hypoxic conditions accelerated the post-exercise return of cTnT to resting values. Furthermore, more severe hypoxia attenuated the cTnT response to exercise—the magnitude of the increase at 2 h was significantly smaller under H3000 than under normoxia (ΔcTnT: N = 53.9% vs. H3000 = 26.3%).

A different response pattern was observed for cTnI. In the UT group, cTnI concentrations increased significantly at 2 h post-exercise, remained elevated at 6 h of recovery, and returned to baseline by 24 h. In the T group, the kinetics of cTnI changes varied depending on the condition. Under normoxia, cTnI increased at 2 h, peaked at 6 h, and remained elevated at 24 h post-exercise. Under H2000, a significant increase in cTnI was observed only at 6 h post-exercise, whereas under H3000, cTnI did not change significantly at any time point. These findings indicate an inhibitory effect of the hypoxic stimulus on the post-exercise cTnI response during exhaustive high-intensity exercise in athletes.

Previous studies have demonstrated that cTnT increases significantly following exercise under both normoxic and hypoxic conditions [15,27], whereas cTnI does not change significantly immediately after exercise under either condition [27]. The differing response patterns of cTnT and cTnI reflect differences in the release kinetics of the two proteins [15,75,76]. The temporally delayed response of cTnI relative to cTnT reflects the slower release of cTnI compared to cTnT [15,75,76]. Klinkenberg et al. [75] showed that cTnT peaked at 2 h, whereas cTnI peaked at 5 h following completion of endurance exercise. Similarly, Skadberg et al. [76] found that cTnT peaked immediately after exercise, while cTnI peaked 3 h after its completion. These findings, together with the results of the present study, indicate that cTnI and cTnT respond differently to physical exercise, with cTnI characterized by slower release kinetics.

The difference between athletes and untrained individuals in the cTnI response to exercise likely reflects long-term training adaptation in the T group, which—through increased structural stability of cardiomyocytes and reduced membrane permeability—limits cTnI efflux into the bloodstream even during high-intensity exercise. Under hypoxic conditions, this effect is further reinforced by the earlier onset of peripheral fatigue, which leads to termination of exercise at a lower absolute mechanical load and thus a reduced injurious stimulus to the myocardium [77].

The considerable interindividual variability in post-exercise troponin release, widely noted in the literature [12,78,79], was also reflected in the findings of the present study. Particularly large dispersion of cTnT values was observed in the UT group, whereas variability in the T group was markedly lower. This observation is consistent with reports indicating that training experience is among the factors influencing post-exercise troponin release [77,80], although the direct effect of training status on reducing interindividual variability in the troponin response warrants further investigation.

### 3.6. N-Terminal Pro-B-Type Natriuretic Peptide (NT-proBNP)

B-type natriuretic peptide (BNP) and its inactive N-terminal fragment (NT-proBNP) are released by cardiomyocytes in response to increased myocardial wall stress. The principal stimuli for BNP and NT-proBNP synthesis and secretion are elevated end-diastolic ventricular pressure, increased circulating blood volume, raised filling pressure, and myocardial ischemia [81]. Both BNP and NT-proBNP are considered to play a cardioprotective role in the heart, regulating filling pressure and modulating myocardial remodeling, making them indicators of hemodynamic overload rather than markers of myocardial injury per se [82].

Endurance exercise under normoxia has been shown to produce a transient increase in blood NT-proBNP concentrations in both healthy untrained individuals and athletes [83,84,85,86]. Vidotto et al. [87] observed that NT-proBNP concentrations increased significantly following endurance competition, subsequently normalizing within 24–48 h. Our findings are consistent with these reports, although the kinetics of NT-proBNP changes differed between untrained individuals and athletes. In athletes under normoxia, we observed a transient increase in NT-proBNP, peaking at 2 h post-exercise and returning to baseline by 24 h. In untrained individuals, NT-proBNP increased immediately after exercise and remained elevated at all subsequent measurement points, including at 24 h post-exercise. It may be speculated that these differences reflect a greater hemodynamic cardiac load in untrained individuals in response to exhaustive exercise. Exercise duration, intensity, and training status have previously been shown to significantly modify the amplitude and kinetics of NT-proBNP elevation [88,89,90].

Hypoxia represents an additional hemodynamic and neurohumoral stimulus influencing NT-proBNP changes [91]. The principal mechanism responsible for the increase in NT-proBNP concentrations under hypoxic conditions is pulmonary vasoconstriction, leading to elevated pulmonary arterial pressure and a secondary increase in right ventricular afterload. Increased right ventricular wall stress stimulates cardiomyocytes to synthesize and release proBNP. A further contributing factor may be hypoxia-induced activation of the sympathetic nervous system and the renin-angiotensin-aldosterone axis, amplifying volume and pressure overload in both cardiac ventricles [92]. Under natural altitude conditions, a height-dependent pattern of NT-proBNP elevation has been observed, increasing proportionally with the degree of hypoxia and the duration of altitude exposure [93].

The combination of acute hypoxia and physical exercise appears to represent a particularly intense stimulus for the cardiovascular system. Woods et al. [16] demonstrated that NT-proBNP concentrations in healthy individuals increased significantly immediately after endurance exercise under normobaric hypoxia (equivalent to 3375 m a.s.l.) and remained significantly elevated 2 h later, whereas exercise under normoxia produced no significant changes in NT-proBNP. Our findings revealed that both normoxic and hypoxic exercise led to increases in NT-proBNP; however, the concentration increases at 2 h and 6 h relative to resting values were lower under hypoxic conditions than under normoxia. Furthermore, we observed that in athletes, NT-proBNP concentrations remained significantly elevated at 6 h post-exercise under normoxia, whereas under hypoxic conditions they had returned to resting values. These findings suggest that hypoxic conditions reduced the hemodynamic load on the myocardium during exhaustive high-intensity exercise, particularly in the athlete group. The discrepancy between our results and those of Woods et al. [16] likely reflects differences in the nature and intensity of the exercise employed. In the Woods et al. [16] study, participants performed 2 h of low-intensity exercise (55% W_max_ adjusted to conditions). In our intervention, exercise was performed at high intensity to volitional exhaustion (GXT + CXT). With this type of exercise, hypoxic conditions—as noted earlier—led to the earlier onset of peripheral fatigue, resulting in termination of exercise at a lower absolute mechanical load and consequently a smaller hemodynamic overload of the heart, reflected in the attenuated NT-proBNP increases.

### 3.7. Practical Applications and Limitations

The findings of the present study indicate that moderate normobaric hypoxia does not exacerbate the transient post-exercise changes in cardiac biomarker concentrations observed following exercise under normoxia. Taken together with our previous echocardiographic studies, in which we demonstrated that hypoxic conditions do not augment post-exercise impairment of right or left ventricular function [25,26], it may be concluded that hypoxia corresponding to altitudes of 2000–3000 m a.s.l. represents a safe stimulus for the performance of high-intensity endurance exercise. This stimulus may be particularly beneficial in athletes, in whom hypoxia reduces myocardial load by inducing earlier peripheral fatigue, leading to termination of exercise at a lower absolute workload.

From a diagnostic perspective, our findings regarding the kinetics of cardiac biomarker changes following exercise suggest that, in order to capture peak concentrations of individual biomarkers, measurements should be taken in the 2–6 h period following exercise, when blood concentrations reach their highest values. An important limitation of using certain cardiac biomarker assays—particularly Mb, H-FABP, CK-MB, and IMA—for the diagnosis of exercise-induced myocardial ischemia in physically active individuals is the relatively low tissue specificity of these markers and their origin in skeletal muscle rather than the myocardium. Furthermore, the practical application of cardiac biomarkers in sports diagnostics requires the development of sport-specific reference values, as interpretation of data using standard reference ranges derived from sedentary populations may be misleading when applied to athletes.

Our study showed that acute, moderate normobaric hypoxia did not exacerbate the post-exercise cardiac marker response in untrained individuals and athletes. In practice, to ensure participant safety, it is advisable to consider minimum health requirements and a medical assessment before initiating hypoxic training. Most current recommendations regarding eligibility for hypoxic training or exposure to hypoxic environments are based on a detailed medical history, cardiovascular risk assessment, resting ECG, and identification of cardiovascular, pulmonary, and neurological contraindications [94]. Recently, hypoxic exercise testing has also been proposed as a tool for predicting altitude-related complications and identifying high-risk individuals before hypoxic training [95,96].

Absolute contraindications include unstable coronary artery disease, recent myocardial infarction, decompensated heart failure, severe cardiac arrhythmias, severe uncontrolled hypertension, unstable angina pectoris, active thromboembolic disease, severe respiratory failure, acute infectious disease, and pregnancy. Relative contraindications include severe anemia, uncontrolled asthma, sleep apnea, previous HAPE or HACE, unstable diabetes mellitus, impaired exercise tolerance, low cardiorespiratory fitness, electrolyte disturbances, active autoimmune disease, and uncontrolled endocrine disorders [94,97].

It should be noted that currently available screening recommendations have primarily been developed for high-altitude exposure and generally refer to altitudes higher than those investigated in the present study. Future research should focus on developing evidence-based screening procedures for individuals undertaking training under varying severity of normobaric hypoxia.

Our study is not without limitations. First, the relatively small sample size limits the generalizability of the findings. As the study population consisted exclusively of healthy men aged 20–40 years, the results cannot be directly extrapolated to women, other age groups, or individuals with comorbidities. Second, although exercise intensity was individually adjusted to ensure comparable metabolic load under normoxic and hypoxic conditions, the mechanical load (power output) achieved under hypoxia was lower due to the physiological constraints associated with reduced oxygen availability. The potential influence of differing exercise durations on cardiac biomarker concentrations and kinetics also cannot be excluded.

## 4. Materials and Methods

### 4.1. Study Participants

The study involved 12 men training in cycling (T group; age: 26.5 ± 7.7 years; height: 180.8 ± 3.6 cm; body mass: 71.4 ± 5.3 kg; body fat: 12.3 ± 2.6%; VO_2max_: 64.2 ± 2.9 mL∙kg^−1^∙min^−1^) and 12 untrained, healthy men (UT group; age: 31.7 ± 8.3 years; height: 181.1 ± 6.5 cm; body mass: 85.7 ± 11.5 kg; body fat: 17.3 ± 6.3%; VO_2max_: 44.1 ± 7.4 mL∙kg^−1^∙min^−1^). An a priori analysis (performed using the software GPower 3.1 [98] showed that, for n = 24, while maintaining an acceptable power (1-β = 0.80) and α = 0.05, the repeated measures ANOVA allowed for detection of an effect size of 0.30 (assuming a drop-out rate of 20%). The participants in the training group met the following criteria: VO_2max_ of not less than 60 mL/kg/min, training experience of at least 6 years and at least a six-month washout period from any previous altitude training. The six-month washout criterion applied to all forms of hypoxic exposure, including normobaric or hypobaric hypoxia at rest or during exercise. One participant in the control group and two in the trained group had prior hypoxic exposure, in each case more than six months before enrolment. Additionally, inclusion criteria in both groups were: (1) age 20–40 years; (2) no chronic diseases; (3) systolic blood pressure 100–140 mmHg and diastolic blood pressure 60–90 mmHg. The study excluded participants who (1) used drugs, consumed alcohol or smoked; (2) had hypertension; (3) prematurely stopped the exercise test. In the trained group, all testing sessions were conducted during the transition phase of the annual training cycle, characterized by the lowest training loads, in order to minimize the potential influence of concurrent training adaptations on the study outcomes.

Before the experiment, a preliminary test was carried out, with all participants performing an incremental test on a cycle ergometer under normoxic conditions (Figure 1). The test determined the VO_2max_, verified meeting the basic criterion for participation in the training group (VO_2max_ not less than 60 mL/kg/min) and familiarized participants with testing protocol. All subjects had up-to-date medical examination certificates confirming their good health status and ability to perform intensive physical exercise. Additionally, all the subjects were informed of the aim of the study and provided written informed consent to participate in the study.

The study adhered to the principles outlined in the Declaration of Helsinki and was approved by the Bioethics Committee at the University of Zielona Góra, Poland (Resolution No. 21/2022 of 9 November 2022). The data presented here are part of a larger research project (ClinicalTrials.gov NCT06896773, approved on 9 January 2025) investigating the cardiovascular response to exhaustive exercise under normobaric hypoxia in trained and untrained men. The same cohort and the same exercise protocol have also been used to assess right ventricular and right atrial function [26] and left ventricular systolic and diastolic function [25] by transthoracic echocardiography under normoxia and at simulated 3000 m altitude. The present manuscript reports an entirely separate dataset focused on the kinetics of cardiac biomarkers measured across three altitude conditions (normoxia, 2000 m, and 3000 m a.s.l.) at five time points up to 24 h post-exercise.

### 4.2. Study Design

**Figure 4:**
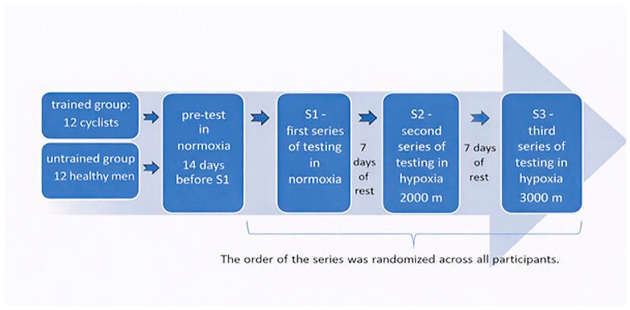
Illustration of the study design.

The experiment consisted of three series differing in terms of simulated altitude: S1 (normoxia), S2 (2000 m a.s.l.), and S3 (3000 m a.s.l.). Each research series consisted of two days of measurements. All participants performed two consecutive cycle ergometer tests with a 10-min active recovery break under different environmental conditions (S1—normoxia, S2—2000 m a.s.l., S3—3000 m a.s.l.). The order of the series was randomized across all participants (Figure 1). All series were performed in a normobaric hypoxic chamber. Participants were not informed about the conditions in the chamber. The fraction of inspired oxygen (FiO_2_) depended on the series and was as follows: S1 (normoxia) = 20.9%; S2 (2000 m a.s.l.) = 16.5%; S3 (3000 m a.s.l.) = 14.4% (Figure 4). The hypoxic environment was maintained using AirZone climate technology (AirSport, Poland). Each series followed the same methodology, and the tests were performed at the same time of the day. Between series, participants observed a one-week active rest period, during which exposure to hypoxia and performance of high-intensity exercise were prohibited.

### 4.3. Testing Protocol

In the first series of testing, before breakfast, body mass and body composition were measured between 7:00 and 7:30 am. Body height was measured using an anthropometer with an accuracy of 0.5 cm, and body composition was estimated using bioelectrical impedance analysis (InBody 220, Biospace, Seoul, South Korea).

**Figure 5:**
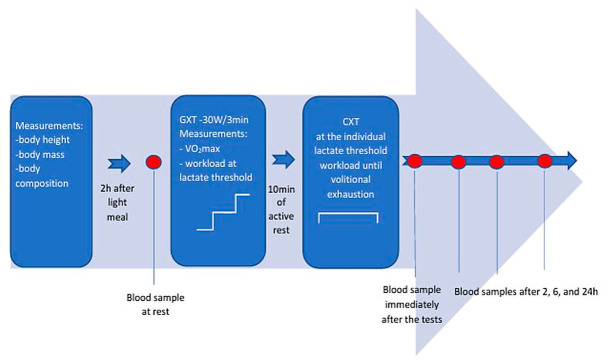
Illustration of the testing protocol during all series of testing.

In all series of testing, two hours after the consumption of a light mixed meal (5 kcal/kg body weight; 50% carbohydrates, 30% fat, 20% protein), all participants performed two consecutive cycle ergometer tests with a 10-min active recovery period (Figure 5). All tests were performed on a cycle ergometer (Excalibur Sport, Lode BV, Groningen, The Netherlands) adjusted individually to each participant (Figure 5).

The first test was a graded exercise test (GXT) until volitional exhaustion. Exercise began at a workload of 90 W, with increments of 30 W every three minutes until volitional exhaustion. Cardiorespiratory variables were continuously recorded at rest and throughout the GXT using a breath-by-breath gas analyzer (MetaLyzer 3B, Cortex, Leipzig, Germany) for the determination of VO_2max_. Additionally, at rest and during the GXT, blood oxygen saturation (SpO_2_) was continuously recorded (WristOx2 3150, Nonin Medical Inc., Plymouth, MN, USA).

When the final stage was not completed, maximal workload (WR_max_) was calculated from the following formula: WR_max_ = WR_k_ + (t/T × WR_p_) [99], where WR_k_ = previous workload, t = exercise duration with the workload until exhaustion, T = duration of each workload, WR_p_ = the workload increment per stage. At the end of each load (last 15 s) capillary blood samples were obtained from the fingertip in order to determine blood lactate levels (LA; SUPER GL2, Dr. Müller Gerätebau GmbH). These data were used to analyze the kinetics of LA concentration in blood and evaluate lactate threshold based on the D_max_ method [100]. This was followed by a 10-min active rest period (30% of the maximum workload reached during the test until volitional exhaustion, WR_max_). Then, the participants performed constant-workload exercise test (CXT) at the lactate threshold workload (WR_LT_) until volitional exhaustion. During the CXT, the participants were allowed to drink water ad libitum. Before and immediately after the CXT, capillary blood was obtained from the fingertip to determine the LA concentration and acid–base balance. Additionally, at rest and during the CXT, HR and SpO_2_ were continuously measured (WristOx2 3150, Nonin Medical Inc., Plymouth, MN, USA).

### 4.4. Blood Sampling

For each participant, 8 mL blood samples were collected from the antecubital vein at five time points during each series of the study: before the exercise tests (after 20-min of passive exposure to the conditions in the chamber), immediately after GXT and CXT, and after 2, 6, and 24 h.

Blood morphology was assessed in EDTA-anticoagulated venous blood on an automated hematology analyzer (ADVIA 60 Siemens Healthineers, Erlangen, Germany). Blood for serum measurements was collected in clot activator tubes. Serum samples were stored at −80 °C for further analysis.

### 4.5. Determination of Cardiac Biomarker Concentration

The following measurements were taken: Cardiac Troponin I and Troponin T (cTnI and cTnT), Myoglobin (Mb), Creatine Kinase MB Isoform (CK-MB), Heart-Type Fatty Acid-Binding Protein (H-FABP), Ischemia-modified Albumin (IMA), and N-Terminal Pro-B-Type Natriuretic Peptide (NT-proBNP). The concentrations of all cardiac biomarkers studied were determined using commercially available ELISA kits (Shanghai Sunred Biological Technology Co.) according to the manufacturer’s instructions after appropriate dilution with the blocking and sample solutions (provided with the kit). The absorbance was measured using a microplate reader (BioTek Instruments, Winooski, VT, USA) set at 450 nm. All cardiac biomarker measurements were performed in duplicate on each sample. Additionally, all cardiac biomarkers were normalized to the corresponding hematocrit value from the same sample [101].

### 4.6. Statistical Analysis

Data were collected and processed using Statistica software v.13 StatSoft, Tulsa, OK, USA). The normality of distribution was assessed using the Shapiro–Wilk test. As most variables violated the assumption of normality, non-parametric tests were applied throughout. Differences across repeated measurements were assessed using the Friedman ANOVA, with the Wilcoxon signed-rank test used for post-hoc comparisons. For all analyses, the statistical significance was set at *p* < 0.05.

## 5. Conclusions

Acute moderate normobaric hypoxia was not found to exacerbate the post-exercise cardiac biomarker response in untrained individuals or athletes. Hypoxia was found to attenuate the increases in Mb, NT-proBNP, and IMA during recovery following exhaustive high-intensity exercise, likely as a consequence of the lower absolute workload resulting from hypoxia-induced earlier peripheral fatigue. The attenuating effect was more pronounced under H3000 than H2000, suggesting a dose-dependent relationship between hypoxic severity and the cardiac biomarker response. Regardless of condition, peak biomarker concentrations were observed at 2–6 h post-exercise in both groups, indicating that single time-point assessment immediately post-exercise is insufficient to capture the full magnitude of the response. Notably, the inhibitory effect of hypoxia on cTnI release was observed exclusively in athletes, suggesting that training-related cardiac adaptations interact with hypoxic conditions to modulate the troponin response. These findings support the safety of exercise training at simulated altitudes of 2000–3000 m a.s.l. and highlight the need for sport-specific reference values for cardiac biomarker interpretation in athletic populations.

## Figures and Tables

**Table 1:** Cardiac marker response to exercise under normoxic and hypoxic conditions (H2000, H3000) in the untrained (UT) group. All values shown as mean ± SD and median (Me).

	Con.	Group UT					
		at Rest	Post ex.	After 2 h	After 6 h	After 24 h	Friedman ANOVA
cTnT (pg/mL)	N	17.51 ± 16.24 Me: 9.77	22.75 ± 20.33 ** Me: 16.12	27.59 ± 25.81 ** Me: 14.92	24.67 ± 21.88 ** Me: 14.13	20.27 ± 18.33 * Me: 13.61	χ^2^(4) = 22.000 *p* = 0.001
	2000	22.10 ± 17.68 Me: 15.87	26.57 ± 21.20 ** Me: 20.59	28.55 ± 21.54 ** Me: 24.01	26.86 ± 20.77 ** Me: 21.38	21.53 ± 17.64 Me: 14.21	χ^2^(4) = 32.666 *p* = 0.001
	3000	24.72 ± 20.48 Me: 17.52	27.91 ± 22.52 ** Me: 21.82	31.31 ± 25.34 ** Me: 24.19	28.22 ± 21.81 Me: 24.02	23.63 ± 19.70 Me: 15.85	χ^2^(4) = 24.533 *p* = 0.001
cTnI (pg/mL)	N	182.52 ± 54.76 Me: 160.41	193.73 ± 64.27 Me: 171.86	223.01 ± 79.69 ** Me:185.14	217.55 ± 73.55 ** Me:193,64	182.19 ± 61.42 Me: 169.79	χ^2^(4) = 17.933 *p* = 0.002
	2000	192.33 ± 57.97 Me: 172.93	205.30 ± 81.97 Me: 170.39	227.53 ± 83.08 ** Me: 197.47	235.03 ± 79.46 ** Me: 197.16	187.80 ± 61.23 Me: 168.01	χ^2^(4) = 26.133 *p* = 0.001
	3000	184.59 ± 64.79 Me: 159.75	193.04 ± 73.48 Me: 158.83	220.85 ± 73.71 ** Me: 185.07	220.91 ± 67.05 ** Me: 203.91	193.60 ± 59.16 Me: 172.52	χ^2^(4) = 23.800 *p* = 0.001
Mb (ng/mL)	N	24.08 ± 17.65 Me: 17.29	32.79 ± 29.37 ** Me: 22.33	39.57 ± 39.24 ** Me: 29.14	29.28 ± 17.88 ** Me: 22.89	28.47 ± 16.36 ** Me: 24.13	χ^2^(4) = 28.333 *p* = 0.001
	2000	25.07 ± 17.33 Me: 19.05	30.43 ± 24.99 ** Me: 22.16	33.76 ± 25.48 ** Me: 26.39	30.07 ± 19.32 ** Me: 25.22	27.46 ± 19.76 Me: 23.21	χ^2^(4) = 27.000 *p* = 0.001
	3000	24.53 ±17.23 Me: 18.16	29.74 ± 25.44 ** Me: 19.31	31.95 ± 26.05 ** Me: 24.53	30.87 ± 17.53 ** Me: 28.36	26.20 ± 17.04 Me: 20.57	χ^2^(4) = 31.666 *p* = 0.001
H-FABP (ng/mL)	N	1.13 ± 0.43 Me: 1.08	1.35 ± 0.62 ** Me: 1.27	1.50 ± 0.61 ** Me: 1.45	1.28 ± 0.65 Me: 1.13	1.28 ± 0.50 Me: 1.09	χ^2^(4) = 16.866 *p* = 0.002
	2000	1.12 ± 0.43 Me: 1.12	1.24 ± 0.48 ** Me: 1.18	1.33 ± 0.55 ** Me: 1.20	1.32 ± 0.60 ** Me: 1.24	1.13 ± 0.34 Me: 1.09	χ^2^(4) = 16.866 *p* = 0.002
	3000	1.13 ± 0.43 Me: 0.99	1.21 ± 0.47 * Me: 1.19	1.31 ± 0.54 ** Me: 1.18	1.23 ± 0.42 Me: 1.23	1.12 ± 0.37 Me: 1.08	χ^2^(4) = 11.800 *p* = 0.018
CK-MB (ng/mL)	N	7.29 ± 3.06 Me: 6.93	7.81 ± 2.93 ** Me: 7.64	8.86 ± 3.28 ** Me: 8.24	9.30 ± 3.91 ** Me: 8.22	7.40 ± 3.28 Me: 7.24	χ^2^(4) = 31.481 *p* = 0.001
	2000	7.02 ± 3.01 Me: 6.28	7.34 ± 2.80 Me: 7.44	8.65 ± 3.68 ** Me: 8.06	9.05 ± 3.16 ** Me: 8.33	7.53 ± 3.10 Me: 6.97	χ^2^(4) = 24.666 *p* = 0.001
	3000	7.02 ± 2.72 Me: 7.18	7.73 ± 2.92 ** Me: 7.68	9.06 ± 3.59 ** Me: 7.64	9.15 ± 3.45 ** Me: 7.97	7.67 ± 2.46 Me: 7.24	χ^2^(4) = 21.266 *p* = 0.001
NT-proBNP (pg/mL)	N	54.91 ± 18.34 Me: 47.83	63.82 ± 25.84 ** Me: 51.72	71.86 ± 22.80 ** Me: 64.57	61.02 ± 22.05 * Me: 49.92	61.22 ± 19.91 * Me: 57.2	χ^2^(4) = 19.533 *p* = 0.001
	2000	52.76 ± 16.61 Me: 48.61	63.36 ± 28.38 ** Me: 49.89	67.15 ± 23.48 ** Me: 57.06	64.76 ± 14.38 * Me: 60.68	59.13 ± 23.21 Me: 57.19	χ^2^(4) = 17.333 *p* = 0.001
	3000	55.86 ± 18.15 Me: 49.22	62.46 ± 27.52 ** Me: 48.73	64.06 ± 21.80 * Me: 55.21	63.53 ± 18.57 * Me: 54.58	63.14 ± 21.36 Me: 60.55	χ^2^(4) = 10.400 *p* = 0.034
IMA (pg/mL)	N	56.90 ± 27.89 Me: 51.80	80.07 ± 32.29 ** Me: 76.65	85.35 ± 22.40 ** Me: 83.28	90.31 ± 42.54 ** Me: 78.13	80.86 ± 36.52 * Me: 76.89	χ^2^(4) = 19.800 *p* = 0.001
	2000	63.84 ± 31.08 Me: 62.84	109.82 ± 58.46 ** Me: 97.88	114.17 ± 52.98 ** Me: 101.36	116.17 ± 36.17 ** Me: 104.56	90.41 ± 30.10 * Me: 82.16	χ^2^(4) = 26.066 *p* = 0.001
	3000	64.33 ± 37.91 Me: 53.89	121.89 ± 50.98 ** Me: 103.21	144.76 ± 62.17 ** Me: 130.58	130.73 ± 54.79 ** Me: 115.52	92.05 ± 51.84 * Me: 84.01	χ^2^(4) = 29.533 *p* = 0.001

Abbreviations: cTnT—Cardiac Troponin T, cTnI—Cardiac Troponin I, Mb—Myoglobin, CK-MB—Creatine Kinase MB Isoform, H-FABP—Heart-Type Fatty Acid-Binding Protein, NT-proBNP—N-Terminal Pro-B-Type Natriuretic Peptide, IMA—Ischemia-modified albumin. ** *p* < 0.01, * *p* < 0.05—significant differences compared to baseline (at rest).

**Table 2:** Cardiac marker response to exercise under normoxic and hypoxic conditions (H2000, H3000) in the trained (T) group. All values shown as mean ± SD and median (Me).

	Con.	Group T					
		at Rest	Post ex.	After 2 h	After 6 h	After 24 h	Friedman ANOVA
cTnT (pg/mL)	N	28.85 ± 17.48 Me: 23.78	36.71 ± 22.84 ** Me: 29.61	38.33 ± 21.14 ** Me: 29.34	37.96 ± 22.58 ** Me: 28.32	32.21 ± 17.35 Me: 24.85	χ^2^(4) = 22.133 *p* = 0.001
	2000	30.34 ± 20.62 Me: 20.89	37.01 ± 22.83 ** Me: 29.48	38.73 ± 22.87 * Me: 29.55	34.84 ± 21.67 * Me: 27.36	28.17 ± 17.71 Me: 21.09	χ^2^(4) = 29.466 *p* = 0.001
	3000	31.29 ± 22.51 Me: 23.69	36.53 ± 26.98 * Me: 25.43	35.43 ± 26.87 * Me: 24.14	35.70 ± 22.33 ** Me: 26.65	30.27 ± 18.14 Me: 23.82	χ^2^(4) = 14.533 *p* = 0.005
cTnI (pg/mL)	N	166.83 ± 33.64 Me: 155.47	171.89 ± 48.38 Me: 159.45	179.98 ± 36.67 * Me: 173.75	201.17 ± 47.44 ** Me: 198.14	187.66 ± 54.64 * Me: 173.20	χ^2^(4) = 14.133 *p* = 0.006
	2000	166.86 ± 52.47 Me: 150.71	165.19 ± 48.42 Me: 147.53	178.85 ± 49.35 Me: 164.85	190.32 ± 46.75 * Me: 174.45	161.81 ± 41.76 Me: 148.22	χ^2^(4) = 18.866 *p* = 0.001
	3000	161.38 ± 53.35 Me: 141.15	164.32 ± 53.43 Me: 145.50	162.28 ± 68.37 Me: 155.42	178.04 ± 52.16 Me: 155.95	164.25 ± 42.97 Me: 148.87	NS
Mb (ng/mL)	N	39.19 ± 44.75 Me: 19.46	43.63 ± 47.08 ** Me: 25.67	55.19 ± 54.53 ** Me: 27.90	56.39 ± 67.76 *** Me: 29.31	39.34 ± 38.76 Me: 24.41	χ^2^(4) = 33.000 *p* = 0.001
	2000	40.21 ± 45.90 Me: 21.04	45.81 ± 54.07 * Me: 26.51	52.41 ± 55.32 ** Me: 28.18	47.11 ± 51.86 ** Me: 25.16	43.88 ± 53.70 Me: 23.55	χ^2^(4) = 24.400 *p* = 0.001
	3000	39.68 ± 44.54 Me: 22.40	43.08 ± 50.27 * Me: 22.49	46.27 ± 47.34 ** Me: 25.06	44.98 ± 47.93 ** Me: 26.09	41.58 ± 44.78 Me: 22.77	χ^2^(4) = 22.533 *p* = 0.011
H-FABP (ng/mL)	N	1.28 ± 0.977 Me: 1.02	1.55 ± 1.31 ** Me: 1.12	1.72 ± 1.31 ** Me: 1.33	1.61 ± 1.34 * Me: 1.20	1.53 ± 1.36 Me: 0.94	χ^2^(4) = 18.533 *p* = 0.001
	2000	1.39 ± 1.15 Me: 1.03	1.49 ± 1.17 * Me: 1.08	1.68 ± 1.33 ** Me: 1.22	1.40 ± 1.23 Me: 0.97	1.32 ± 1.05 Me: 0.98	χ^2^(4) = 18.866 *p* = 0.001
	3000	1.31 ± 1.03 Me: 0.93	1.55 ± 1.27 Me: 1.14	1.65 ± 1.33 * Me: 1.12	1.38 ± 1.15 Me: 1.14	1.27 ± 1.04 Me: 0.96	χ^2^(4) = 13.004 *p* = 0.011
CK-MB (ng/mL)	N	7.02 ± 1.77 Me: 7.01	7.55 ± 1.93 ** Me: 8.06	8.34 ± 2.11 ** Me: 8.83	8.83 ± 2.44 ** Me: 8.81	7.23 ± 2.46 Me: 7.19	χ^2^(4) = 21.800 *p* = 0.001
	2000	6.64 ± 2.43 Me: 7.01	7.31 ± 2.15 ** Me: 7.44	7.66 ± 2.42 * Me: 7.63	7.94 ± 2.79 * Me: 7.57	5.78 ± 2.59 Me: 4.51	χ^2^(4) = 22.309 *p* = 0.001
	3000	6.48 ± 2.32 Me: 6.46	7.09 ± 2.24 * Me: 6.63	7.51 ± 2.52 ** Me: 7.41	8.03 ± 2.60 ** Me: 8.51	6.47 ± 1.84 Me: 6.47	χ^2^(4) = 25.600 *p* = 0.001
NT-proBNP (pg/mL)	N	31.17 ± 5.81 Me: 30.28	32.47 ± 6.48 Me: 31.76	38.84 ± 7.37 ** Me: 39.22	36.06 ± 5.61 * Me: 36.87	34.69 ± 7.28 Me: 35.14	χ^2^(4) = 19.066 *p* = 0.001
	2000	31.79 ± 4.10 Me: 30.31	31.98 ± 5.50 Me: 30.29	37.08 ± 4.63 ** Me: 36.58	31.31 ± 5.96 Me: 29.63	32.93 ± 6.93 Me: 33.09	χ^2^(4) = 22.133 *p* = 0.001
	3000	30.25 ± 5.38 Me: 28.35	31.90 ± 4.68 Me: 31.90	35.55 ± 5.41 ** Me: 35.21	28.93 ± 5.32 Me: 29.35	29.40 ± 3.76 Me: 29.59	χ^2^(4) = 15.66 *p* = 0.003
IMA (pg/mL)	N	49.91 ± 43.55 Me: 30.29	61.10 ± 53.36 ** Me: 39.84	69.97 ± 65.79 ** Me: 41.43	73.84 ± 66.03 ** Me: 48.50	56.97 ± 40.71 * Me: 35.63	χ^2^(4) = 35.266 *p* = 0.008
	2000	48.52 ± 44.68 Me: 25.58	58.37 ± 50.75 ** Me: 31.72	71.75 ± 68.15 ** Me: 42.54	72.14 ± 56.60 ** Me: 52.29	53.16 ± 56.22 Me: 30.97	χ^2^(4) = 35.333 *p* = 0.001
	3000	46.48 ± 39.33 Me: 26.42	62.35 ± 51.66 ** Me: 36.38	65.24 ± 50.51 ** Me: 42.17	68.58 ± 51.45 ** Me: 49.42	43.27 ± 34.68 Me: 27.14	χ^2^(4) = 32.800 *p* = 0.001

Abbreviations: cTnT—Cardiac Troponin T, cTnI—Cardiac Troponin I, Mb—Myoglobin, CK-MB—Creatine Kinase MB Isoform, H-FABP—Heart-Type Fatty Acid-Binding Protein, NT-proBNP—N-Terminal Pro-B-Type Natriuretic Peptide, IMA—Ischemia-modified Albumin. NS—Non-significant. *** *p* < 0.001, ** *p* < 0.01, * *p* < 0.05—significant differences compared to baseline (at rest).

**Table 3:** Mechanical work, heart rate, saturation of hemoglobin and ∆ values of lactate and pH during exercise to exhaustion under all conditions (normoxia and hypoxia—H2000, H3000) in the untrained (UT) and trained (T) group. All values shown as mean ± SD and median (Me).

Variable	Group	Normoxia	Hypoxia (2000 m)	Hypoxia (3000 m)
W_mech_ (kJ)	UT	534.6 ± 173.9 Me: 522.6	519.8 ± 169.5 ** Me: 537.6	434.9 ± 144.3 **$$ Me: 408.2
	T	937.8 ± 183.1 ### Me: 958.8	883.4 ± 171.7 ### Me: 885.3	762.1 ± 165.5 *### Me: 766.8
HR_end_ (bpm)	UT	172.4 ± 8.7 Me: 171.0	167.8 ± 8.6 Me: 166.0	164.4 ± 7.6 ** Me: 165.0
	T	179.3 ± 7.4 # Me: 181.0	172.2 ± 10.2 * Me: 172.0	171.7 ± 8.5 ** Me: 173.0
SpO_2end_ (%)	UT	96.2 ± 1.1 Me: 96.0	89.8 ± 2.5 * Me: 90.0	84.9 ± 2.4 **$$ Me: 85.0
	T	94.2 ± 1.1 ### Me: 94.0	89.4 ± 2.3 ** Me: 90.0	84.4 ± 4.4 **$ Me: 85.5
∆LA (mmol/L)	UT	6.52 ± 1.90 Me: 6.26	6.39 ± 1.46 Me: 6.42	6.36 ± 2.36 Me: 5.71
	T	5.18 ± 1.72 Me: 5.00	4.95 ± 2.24 Me: 3.96	4.76 ± 1.72 Me: 4.14
∆pH	UT	−0.114 ± 0.048 Me: −0.107	−0.092 ± 0.046 Me: −0.088	−0.101 ± 0.041 Me: −0.106
	T	−0.091 ± 0.026 Me: −0.082	−0.080 ± 0.058 Me: −0.068	−0.075 ± 0.042 Me: −0.062

Abbreviations: W_mech_—total mechanical work performed during both tests (GXT + CXT), HR_end_—heart rate recorded at the end of exercise performed to exhaustion, SpO_2end_—hemoglobin saturation registered at the end of exercise, ∆LA—change in blood lactate concentration in response to exercise, ∆pH—change in pH in response to exercise; ** *p* < 0.01, * *p* < 0.05—significant differences between normoxia and hypoxia; $$ *p* < 0.01, $ *p* < 0.05—significant differences between 2000 m and 3000 m; ### *p* < 0.001, # *p* < 0.05—significant differences between groups (UT vs. T) under given conditions. Note: Exercise responses for the normoxia and 3000 m conditions have been reported previously [25,26]; data for the 2000 m condition are reported here for the first time.

## Data Availability

The original contributions presented in this study are included in the article. Further inquiries can be directed to the corresponding author.

## Abbreviations

RV	Right ventricle
PAP	Pulmonary arterial pressure
LV	Left ventricular
cTnT	Cardiac troponin T
H-FABP	Heart-type fatty acid-binding protein
cTnI	Cardiac troponin I
Mb	Myoglobin
CK-MB	Creatine Kinase MB Isoform
IMA	Ischemia-modified Albumin
NT-proBNP	N-Terminal Pro-B-Type Natriuretic Peptide
W_mech_	Total mechanical work performed during both tests
HR_end_	Heart rate recorded at the end of exercise performed to exhaustion
SpO_2end_	Hemoglobin saturation registered at the end of exercise
∆LA	Change in blood lactate concentration in response to exercise,
∆pH	Change in pH in response to exercise
WR_max_	Maximal workload
VO_2max_	Maximal oxygen uptake
GXT	Graded exercise test
SpO_2_	Blood oxygen saturation
CXT	Constant-workload exercise test
